# Controlling Confounding in a Study of Oral Anticoagulants: Comparing Disease Risk Scores Developed Using Different Follow-Up Approaches

**DOI:** 10.5334/egems.254

**Published:** 2019-07-15

**Authors:** Justin Bohn, Sebastian Schneeweiss, Robert J. Glynn, Sengwee Toh, Richard Wyss, Rishi Desai, Joshua J. Gagne

**Affiliations:** 1 Division of Pharmacoepidemiology and Pharmacoeconomics, Department of Medicine, Brigham and Women’s Hospital and Harvard Medical School, Boston, MA, US; 2 Department of Population Medicine, Harvard Medical School and Harvard Pilgrim Health Care Institute, Boston, MA, US

**Keywords:** Health insurance claims data, confounding control, disease risk scores, prognostic scores, comparative effectiveness research

## Abstract

**Purpose:**

Little is known about how disease risk score (DRS) development should proceed under different pharmacoepidemiologic follow-up strategies. In an analysis of dabigatran vs. warfarin and risk of major bleeding, we compared the results of DRS adjustment when models were developed under “intention-to-treat” (ITT) and “as-treated” (AT) approaches.

**Methods:**

We assessed DRS model discrimination, calibration, and ability to induce prognostic balance via the “dry run analysis”. AT treatment effects stratified on each DRS were compared with each other and with a propensity score (PS) stratified reference estimate. Bootstrap resampling of the historical cohort at 10 percent–90 percent sample size was performed to assess the impact of sample size on DRS estimation.

**Results:**

Historically-derived DRS models fit under AT showed greater decrements in discrimination and calibration than those fit under ITT when applied to the concurrent study population. Prognostic balance was approximately equal across DRS models (–6 percent to –7 percent “pseudo-bias” on the hazard ratio scale). Hazard ratios were between 0.76 and 0.78 with all methods of DRS adjustment, while the PS stratified hazard ratio was 0.83. In resampling, AT DRS models showed more overfitting and worse prognostic balance, and led to hazard ratios further from the reference estimate than did ITT DRSs, across sample sizes.

**Conclusions:**

In a study of anticoagulant safety, DRSs developed under an AT principle showed signs of overfitting and reduced confounding control. More research is needed to determine if development of DRSs under ITT is a viable solution to overfitting in other settings.

## Introduction

Disease risk scores (DRSs) have a long history in the confounding control literature [1] and are increasingly being used for confounding adjustment in pharmacoepidemiological studies [2,3,4,5,6,7,8,9,10]. Unlike the more commonly-used propensity score (PS), which models the probability of treatment conditional on confounders, the DRS, or prognostic score, models the expected outcome under the comparator treatment conditional on the confounders. When PS modeling is difficult, such as in studies with small numbers in one treatment group, DRS modeling can provide an alternative dimension-reduction method when the number of confounders is large [3,4].

Hansen developed the theoretical framework for DRSs, showing that their ability to reduce or remove confounding is based on the ability to induce “prognostic balance”, an independence between the outcome that would be observed under the comparator treatment and the confounders, conditional on the DRS [11]. In contrast, conditioning on the PS induces “propensity balance”, an independence between the study treatment and confounders. Hansen compares prognostic and propensity balance to the type of balance that is obtained in laboratory research and clinical trials, respectively. The prognostic balance that results from conditioning on the DRS mimics the tight control of experimental conditions, while the propensity balance that results from conditioning on the PS mimics randomization [2].

Considerable thought has been given to methods for DRS development, including questions of the appropriate time period (i.e., use of historical or concurrent data) and treatment group (i.e., use of the total population or of the comparator population only) for estimation [7] and how to select variables to enter the model [12]. However, little attention has been given to the question of how DRS development should proceed under different models of follow-up for the study outcome.

Commonly, pharmacoepidemiological studies employ one of two follow-up strategies: “intention-to-treat” (ITT) or “as-treated” (AT). The ITT strategy follows patients from therapeutic initiation to the occurrence of an outcome event or loss to follow-up, treating treatment status as fixed at baseline and ignoring post-initiation adherence. In contrast, the AT strategy follows patients until outcome event occurrence, loss to follow-up, or discontinuation of the initial treatment, whichever comes first. This terminology differs from that used in the clinical trial literature, where the approach we describe as AT would often be described as “per-protocol” [13], however we retain the ITT/AT nomenclature for consistency with previous pharmacoepidemiological studies.

The choice of ITT or AT follow-up for the study outcome is generally dictated by the research question at hand, but it should be noted that these strategies yield treatment effect estimates representing different causal contrasts, which are subject to different sources of bias [13]. The choice of which approach to use when developing a DRS is less studied, but investigators will likely mirror the approach used for the main outcome analysis. However, should the investigator decide to estimate a so-called “as-treated effect”, the follow-up of patients for DRS development under the same AT strategy may introduce practical issues that arise due to a reduction in the number of observed outcome events that are currently not well understood. In this paper, we explore the issue of overfitting DRS models fit under AT follow-up, propose a potential practical solution, and assess its performance in the setting of comparative safety of oral anticoagulants.

Under AT, both the total accrued person-time and number of outcome events observed will be lower than in the ITT approach, and the extent of this decrease will depend on the extent and timing of treatment discontinuation. This in turn has implications for the development of DRS models, since model dimensionality, or correspondingly model goodness of fit, will be affected by the number of events available [14]. Therefore, if the desired final analysis is to be performed under AT, it is possible that one may be unable to obtain robust estimates of DRS model coefficients using an AT approach due to low event counts. This is likely problematic in pharmacoepidemiological studies, which generally rely on proxy adjustment for a large number of confounders defined in secondary data sources [15] and may deal in infrequent clinical outcomes.

One potential solution to this problem is to develop the DRS under an ITT strategy, allowing events occurring after the discontinuation of treatment to be included. This may solve the issue of event scarcity, but at a cost of misspecification of the true target of estimation: the patient-specific expected outcome *that would occur while taking the comparator treatment*, conditional on the confounders. While an ITT DRS may not represent the correct DRS with which to adjust an AT treatment effect, such a DRS may facilitate better confounding control than an overfit AT DRS, or a robust AT DRS that omits relevant confounders. That is, a good estimate of the risk among *initiators* of the comparator treatment may be better than a bad estimate of the risk among *current users of* the comparator treatment.

In order to inform DRS practice in a comparative safety and effectiveness setting, we sought to compare the ability of DRS models developed under ITT and AT approaches to control confounding in a comparison of dabigatran vs. warfarin for the risk of major bleeding events. More specifically, we sought to: (a) describe how different follow-up strategies impact the discrimination and calibration of DRS models, (b) assess DRS models for prognostic balance after fitting under various follow-up strategies, (c) determine the impact of follow-up strategy choice on DRS-based confounding control, and (d) use resampling to explore the impact of sample size on DRS-based confounding control. Finally, in light of these results, we describe the assumptions inherent in choosing different follow-up strategies for DRS development.

## Methods

### Study population

We identified all new users of dabigatran and warfarin in the Truven Marketscan Commercial Database between October 2010 (the month of dabigatran’s approval in the US) and December 2013. At the time of initiation, eligible patients were required to: (a) have been continuously enrolled in their health plan for at least 365 days, (b) be 18 years of age or older, (c) have documented evidence of atrial fibrillation, (d) have a CHA_2_DS_2_-VASc score of at least 1, and (e) have no recorded dispensing of an oral anticoagulant in the preceding 365 days. Patients were excluded if they: (a) were missing information on age and sex, (b) initiated both warfarin and dabigatran on the same date, (c) had a recorded nursing home stay within the 365 days before initiation, or (d) had documented evidence of valvular disease. We refer to this cohort as the “concurrent cohort”. We additionally enumerated a cohort of individuals initiating warfarin between January 2009 and September 2010 for disease risk score estimation, which we refer to as the “historical cohort”. All inclusion/exclusion criteria for this group were the same as those for the concurrent cohort. Follow-up for both cohorts ended in December of 2013.

In both the concurrent and historical cohorts, we used claims from the 365 days preceding initiation to define 69 confounders measuring various aspects of demographics, comorbidity, concomitant and historical medication use and health care utilization. Clinical risk scores including the HAS-BLED [16], CHA_2_DS_2_-VASc [17], and CHADS_2_ [18] were likewise defined with limited adaptations for use with claims data and included as covariates. Counting multi-level categorical variables (and the categorization of continuous measures such as days hospitalized), these covariates accounted for 91 total model terms.

### Cohort follow-up

**Figure 1:**
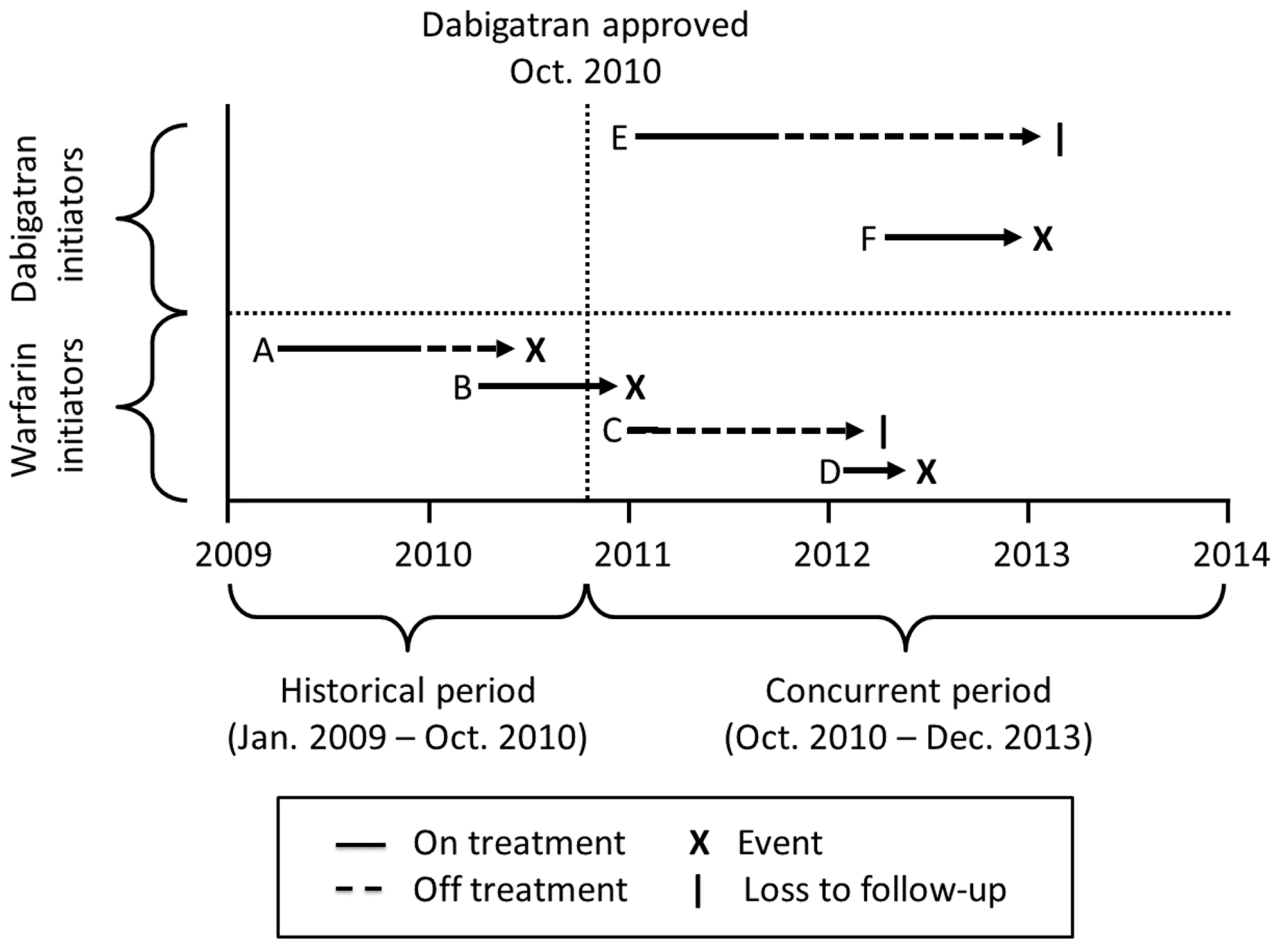
**Follow-up of patients in the study population.** Schematic representation of follow-up in the study population, showing historical warfarin initiators A and B, concurrent warfarin initiators C and D, and concurrent dabigatran initiators E and F. Arrows indicate the duration of observation, becoming dashed when a patient discontinues their index treatment. Events are represented by an “X”, and censoring by a “|”. The disease risk score model is fit among patients A and B only. Note that under “as-treated” follow-up observation stops at discontinuation, and thus only patients B, D, and F have events that are observed.

The outcome of interest was the occurrence of any major bleeding event, excluding hemorrhagic stroke. In both the concurrent and historical cohorts, we followed patients under two broad strategies. Under ITT, patients were followed from the day after treatment initiation until the occurrence of a major bleeding event, study termination (December 31, 2013), or end of enrollment, whichever came first. Under AT, patients were additionally censored upon discontinuation of the index treatment (either due to switching treatments or stopping). We also followed patients under three time-limited ITT approaches, censoring patients at 180, 365, and 730 days of follow-up respectively. Figure 1 outlines the general follow-up strategy.

### Disease risk score development

All DRSs were estimated within the historical cohort of warfarin initiators. While estimation of DRSs within the full concurrent cohort [3], or among only warfarin initiators in the concurrent cohort, have been proposed, historical estimation has several advantages, including the reduced potential for overfitting [2,7] and for bias amplification [19]. Due to patients’ variable lengths of follow-up, we chose to fit Cox proportional hazards models to estimate DRSs, including terms for the 91 main effects of the covariates. Five separate models were fit, following patients for occurrence of the major bleeding outcome under each of the five follow-up strategies: AT, 6-month ITT, 1-year ITT, 2-year ITT, and full ITT. Using the coefficients from these five models, we defined DRSs on the linear predictor scale for patients in both the concurrent and historical cohorts.

### Disease risk score assessment

The discriminatory ability of the DRS models was assessed by the concordance index described by Harrell [20]. In survival analysis, the “C-index” is, informally, the proportion of all pairs of subjects in which the subject with the higher predicted survival actually survived longer, excluding pairs in which both subjects are censored or one is censored before the other fails. Model calibration was assessed by calibration plots constructed with the modified Greenwood-Nam-D’Agostino (G-N-D) method comparing unadjusted Kaplan-Meier risk estimates, to the DRS model average predicted risk at 180 days, within deciles of the DRS linear predictor [21]. For all models, discrimination and calibration were assessed in both the historical and concurrent cohorts with the AT outcome serving as the reference.

We also utilized the “dry run analysis” framework proposed by Hansen to assess the ability of a DRS model to induce prognostic balance [11,22]. The dry run procedure involves sampling the untreated (i.e., concurrent warfarin) population based on their PS to form “pseudo-treated” and “pseudo-untreated” groups of untreated individuals with differences in covariate distributions similar to those of the actual treated and untreated groups. An effect of “pseudo-treatment” can then be estimated comparing these groups, conditional on the DRS. If the DRS induces prognostic balance and the propensity score is correctly specified then this treatment effect must be null since both groups are actually untreated. Hansen therefore argues for using the divergence of this estimate from its null value, which he terms the “pseudo-bias”, as a diagnostic for DRSs that can be performed without reference to the planned, full cohort treatment effect estimate. More information on the dry run approach can be found in Appendix 1.

### Treatment effect estimation

In order to compare rates of major bleeding between concurrent dabigatran and warfarin users, we estimated AT hazard ratios using Cox proportional hazards models, with stratification on deciles of the five DRSs. No DRS trimming was performed. Throughout, we used as a reference estimate the AT hazard ratio comparing dabigatran to warfarin initiators, stratified on deciles of the PS. The same set of covariates were included in the PS and DRS models, with the exception of two variables (history of acute renal disease and number of prothrombin tests ordered) omitted from the PS model for extreme negative associations with dabigatran initiation.

### Resampling study

To assess the impact of sample size on DRS development, we resampled the historical cohort 1,000 times each at 6 sample sizes: 10 percent, 25 percent, 50 percent, 75 percent, and 90 percent of its full sample size. All sampling was done with replacement. In each resampled historical cohort, we fit the five aforementioned DRS models in the manner described above, using the same covariates and methodology. Coefficients from these models were used to define DRSs on the linear predictor scale in both the given resampled historical cohort, and in the concurrent cohort (which was not resampled). For each DRS model in each resampled cohort, we recorded: (a) the number of major bleeding events in the resampled historical cohort, (b) the model C-Index as assessed in the resampled historical cohort, (c) the model C-Index for each treatment group as assessed in the concurrent cohort, and (d) the estimated AT hazard ratio stratified on deciles of the given DRS in the concurrent cohort. We also estimated a dry run “pseudo-bias” for each DRS in each resampled cohort using the dry run approach described in Appendix 1. In this case, only a single dry-run “pseudo-bias” was estimated per resampled historical cohort, since performing a large number of dry run resamples for every resampled DRS model would be computationally prohibitive.

## Results

### Patient characteristics and event rates

There were 79,265 patients followed for a total of 66,109 person-years in the concurrent cohort, of whom 22,809 (28 percent) initiated dabigatran. Among historical initiators of warfarin, there were 3,936 major bleeding events (48 events per 1,000 person-years) observed, which was reduced to 1,059 (48 events per 1,000 person-years) after AT restrictions were applied (Table 1). The incidence of major bleeding events was consistently higher among concurrent initiators of warfarin than among concurrent initiators of dabigatran or historical initiators of warfarin. Table 2 shows the distribution of selected covariates among dabigatran and warfarin initiators in the concurrent and historical cohorts. Dabigatran initiators tended to be younger than warfarin initiators, with lower HASBLED and CHA_2_DS_2_-VASc scores, less renal dysfunction, less coronary artery disease and less upper GI bleeding. Warfarin initiators in the concurrent period tended to be older and less healthy than those in the historical period.

**Table 1:** Incidence of major bleeding under different follow-up approaches, by treatment initiation and period.

	Historical cohort		Concurrent cohort			

	Warfarin		Warfarin		Dabigatran	

Follow-up	Events	IR	Events	IR	Events	IR

AT	1,059	61	1,438	78	430	48
ITT (6-Month)	1,167	65	1,774	79	457	47
ITT (1-Year)	1,933	57	2,505	70	690	43
ITT (2-Year)	3,075	51	2,930	64	803	40
ITT(All)	3,936	48	2,944	64	803	40

Abbreviations: IR, incidence rate per 1,000 person-years; AT, as-treated; ITT, intention-to-treat.

**Table 2:** Selected baseline characteristics by treatment initiation and period.

	Historical cohort	Concurrent cohort	

	Warfarin (N = 39,209)	Warfarin (N = 56,456)	Dabigatran (N = 22,809)

Age, mean (sd)	69.1 (12.4)	71.1 (12.1)	67.3 (12.2)
Male sex, n (%)	23,747 (61)	34,227 (61)	14,600 (64)
Census region, n (%)			
Northeast	6,659 (17)	7,974 (14)	3,495 (15)
South	11,720 (30)	14,361 (25)	7,414 (33)
Midwest	13,076 (33)	12,992 (23)	4,555 (20)
West	7,754 (20)	21129 (37)	7,345 (32)
HASBLED Score, mean (sd)	2.27 (1.01)	2.48 (1.10)	2.18 (1.02)
CHA_2_DS_2_-VASc score, mean (sd)	3.22 (1.59)	3.56 (1.67)	2.91 (1.57)
Renal dysfunction, n (%)	4,775 (12)	10,385 (18)	2,146 (9)
Coronary artery disease, n (%)	12,342 (32)	19,717 (35)	6,768 (30)
Upper GI bleed, n (%)	243 (0.6)	345 (0.6)	62 (0.3)

### Discrimination

All DRS models showed moderate discriminatory ability among the historical warfarin initiators that comprised the DRS development cohort (C-index 0.653–0.678, Table 3). When the DRS was applied in the concurrent cohort, C-indices were consistently higher among dabigatran initiators than among warfarin initiators. The DRS model “optimism” (the difference between the discriminatory ability measured in the historical warfarin initiators and the concurrent warfarin initiators, was lowest for the model developed under ITT and highest for the model developed under AT.

**Table 3:** Discrimination and prognostic balance of disease risk score models, by period and treatment status.

	C-Index					Pseudo-bias		

	Historical cohort	Concurrent cohort						

Follow-up	Warfarin	Warfarin	Dabigatran	All	Optimism	Estimate	95%	CI

AT	0.678	0.622	0.660	0.638	0.056	–6%	–16%	5%
ITT (6-Month)	0.677	0.630	0.676	0.649	0.047	–6%	–15%	5%
ITT (1-Year)	0.666	0.624	0.674	0.643	0.042	–6%	–16%	4%
ITT (2-Year)	0.658	0.621	0.677	0.641	0.037	–7%	–17%	4%
ITT (All)	0.653	0.619	0.683	0.641	0.034	–6%	–15%	4%

Abbreviations: DRS, disease risk score; AT, as-treated; ITT, intention-to-treat; HR, hazard ratio; CI, confidence interval. Footnotes: a) DRS model optimism is the difference between the C-Index for the model in the historical cohort and the C-Index for the model among warfarin initiators in the concurrent cohort. All C-indices were estimated within the AT follow-up experience. b) The dry run “pseudo-bias” is the ability of a DRS to induce prognostic balance. These are presented on the scale of percent bias in the hazard ratio. Values closer to 0% indicate better prognostic balance.

### Calibration

**Figure 2:**
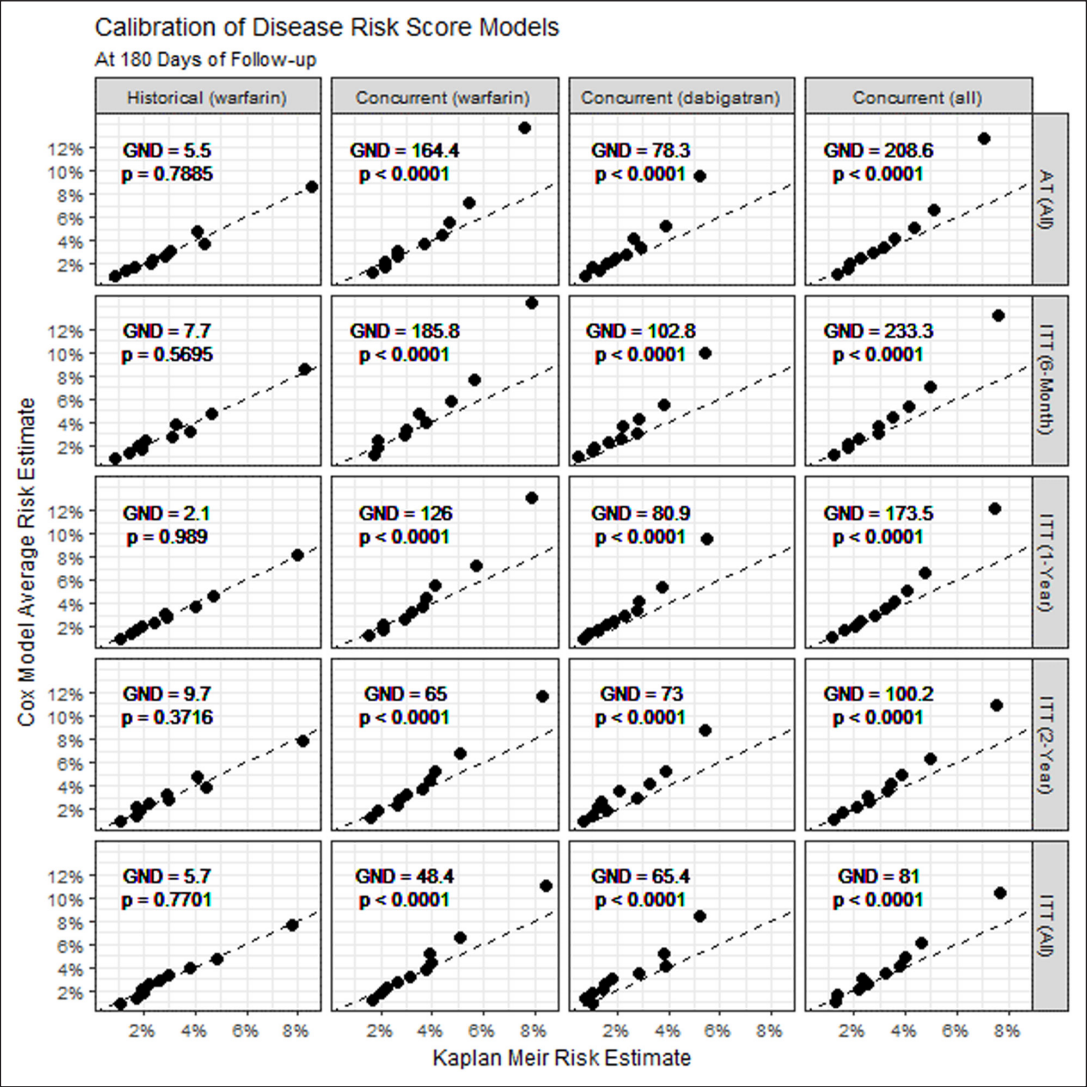
**Calibration of DRS models at 180 days.** Calibration plots showing Kaplan-Meier predicted risk vs. DRS model predicted risk within deciles of the DRS. Calibration is shown at 180 days among historical warfarin initiators, concurrent warfarin initiators, concurrent dabigatran initiators, and the entire concurrent population in columns 1–3, and for the five DRS models (AT, 6-month ITT, 1-year ITT, 2-year ITT, and unlimited ITT) in rows 1–5, respectively. Each point represents a sample decile of the DRS. Dashed lines represent a slope of 1, or exact calibration. P-values and chi-squared test statistics for the Greenwood-Nam-D’Agostino test of the null hypothesis of “no lack of fit” are given at the top of each pane.

Among historical warfarin initiators, DRS model-predicted 180–day risk was close to observed risk in the models developed under all strategies, indicating acceptable calibration with nominally insignificant G-N-D tests throughout (Figure 2). When assessed in the concurrent cohort, there was evidence of overestimation of risk, particularly in the upper two deciles of the DRS, across all models, and all G-N-D tests were highly significant at the 0.001 level. This overestimation was more severe among concurrent warfarin initiators than among concurrent dabigatran initiators, and was greater for the model developed under AT than the model developed under ITT.

### Pseudo-bias

The “pseudo-bias” from the dry run analysis was between –6 percent and –7 percent for all DRS models, with confidence intervals overlapping the null value (0 percent), indicating that some downward bias is likely to remain when conditioning on any of the five DRSs.

### Measures of association

The crude AT hazard ratio was 0.64 (95 percent CI 0.57, 0.71). With stratification on deciles of the DRS, the AT hazard ratios were 0.76 (95 percent CI: 0.68, 0.85) for the AT DRS, 0.78 (95 percent CI: 0.70, 0.87) for the 6-month ITT DRS, 0.77 (95 percent CI: 0.69, 0.86) for the 1-year ITT DRS, 0.76 (95 percent CI: 0.68, 0.85) for the 2-year ITT DRS, and 0.77 (95 percent CI: 0.69, 0.86) for the unlimited ITT DRS. In contrast, the reference AT hazard ratio from an analysis with stratification on deciles of the PS, was 0.83 (95 percent CI: 0.74, 0.93).

### Resampling smaller historical cohorts

**Figure 3:**
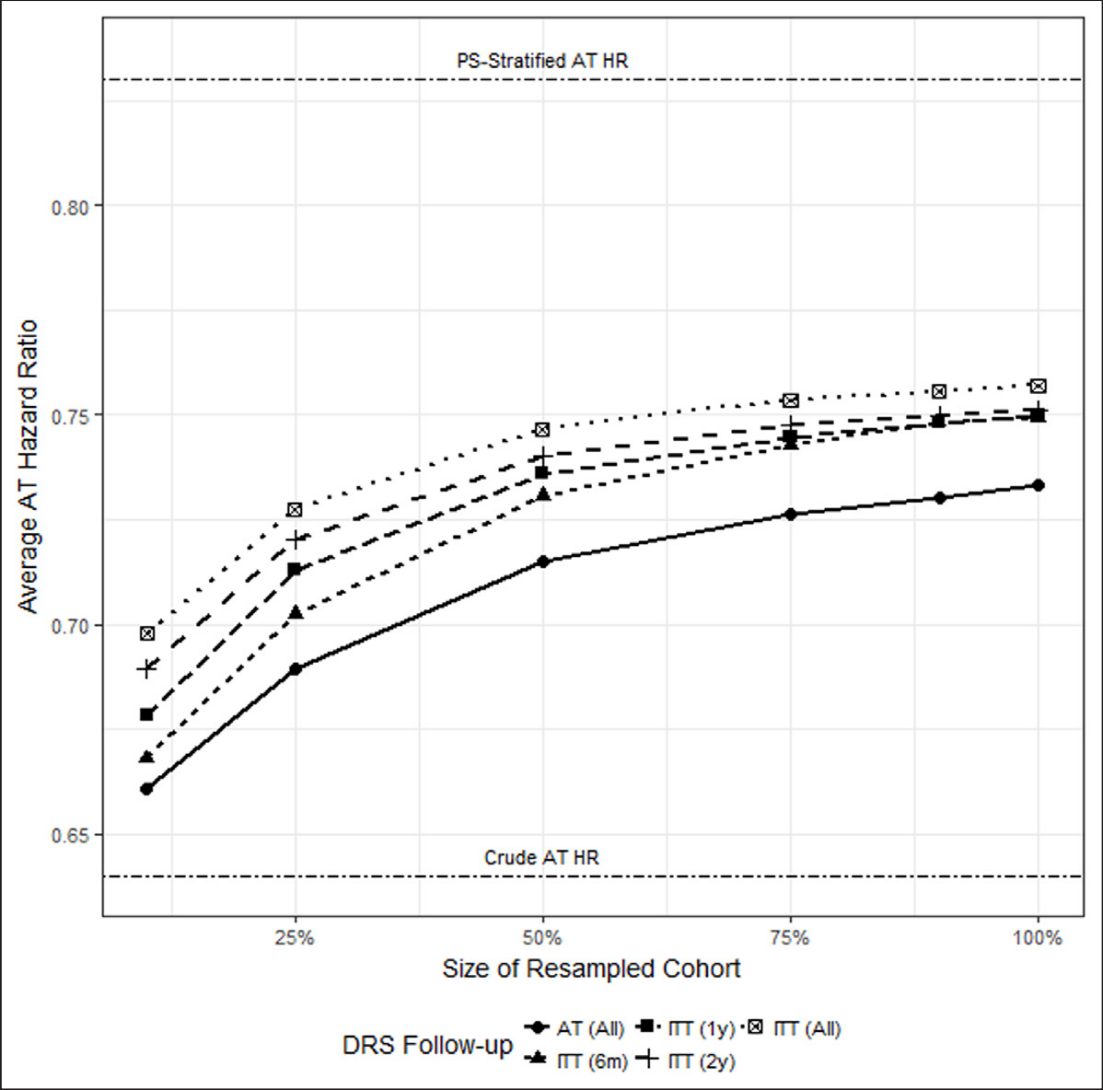
**AT hazard ratios stratified by deciles of five DRSs, averaged across 1,000 models fit to resampled cohorts from 10%–100% sample size.** Estimated as-treated (AT) hazard ratio (HR) vs. sample size, averaged across estimates stratified on deciles of DRS from models fit to 1,000 resampled cohorts at 10%, 25%, 50%, 75%, 90%, and 100% of the full historical cohort sample size. For reference, the crude AT HR and PS-stratified AT HR are presented below and above the plotted curves, respectively.

Across DRS follow-up approaches in the resampling study, DRS model optimism decreased predictably with increasing events-per-variable (Table 4), and was greater for models developed under AT than those developed under ITT. Dry run “pseudo-biases” ranged between –19 percent and –8 percent and decreased with increasing sample size. “Pseudo-bias” values were also consistently closer to the null value of 0 percent bias for the ITT DRS than for the AT DRS, with time-limited ITT DRS values falling in between. Likewise, across historical cohort sample sizes, stratification on the ITT DRS led to more adjustment away from the crude estimate and toward the reference estimate than did stratification on the AT DRS (Figure 3). For the time-limited ITT DRS approaches, the amount of adjustment toward the reference estimate was between that of the AT DRS and the full ITT DRS, with longer follow-up leading to more adjustment.

**Table 4:** Disease risk score discrimination and prognostic balance in the resampling study.

Follow-up	Size^a^	Events^b^	C-Index				Pseudo bias^g^		

			Historical cohort	Concurrent cohort					

			Warfarin^c^	Warfarin^d^	Dabigatran^e^	All^f^	Estimate	95% Empirical CI	

AT (All)	10%	105	0.787	0.524	0.538	0.531	–19%	–28%	–9%
	25%	265	0.729	0.568	0.590	0.579	–15%	–25%	–4%
	50%	529	0.707	0.593	0.621	0.606	–13%	–23%	–1%
	75%	794	0.698	0.602	0.633	0.616	–11%	–22%	1%
	90%	954	0.695	0.605	0.636	0.619	–11%	–21%	2%
ITT (6m)	10%	116	0.765	0.540	0.556	0.547	–18%	–27%	–8%
	25%	292	0.718	0.583	0.612	0.596	–14%	–24%	–3%
	50%	584	0.701	0.606	0.640	0.621	–11%	–22%	0%
	75%	874	0.693	0.613	0.651	0.629	–9%	–21%	2%
	90%	1,050	0.690	0.616	0.656	0.633	–9%	–20%	3%
ITT (1y)	10%	193	0.736	0.552	0.576	0.562	–17%	–26%	–6%
	25%	483	0.698	0.590	0.626	0.605	–13%	–23%	–1%
	50%	965	0.684	0.607	0.649	0.624	–10%	–22%	1%
	75%	1,450	0.678	0.613	0.657	0.630	–9%	–21%	3%
	90%	1,739	0.676	0.615	0.660	0.632	–9%	–19%	3%
ITT (2y)	10%	307	0.708	0.568	0.600	0.580	–16%	–26%	–5%
	25%	769	0.680	0.597	0.640	0.613	–12%	–22%	–1%
	50%	1,537	0.671	0.609	0.658	0.627	–10%	–20%	1%
	75%	2,305	0.667	0.613	0.665	0.632	–9%	–20%	3%
	90%	2,768	0.665	0.614	0.667	0.633	–9%	–19%	3%
ITT (All)	10%	393	0.694	0.573	0.612	0.587	–15%	–25%	–3%
	25%	984	0.671	0.598	0.649	0.616	–11%	–22%	0%
	50%	1,967	0.664	0.608	0.666	0.629	–9%	–20%	2%
	75%	2,951	0.660	0.612	0.672	0.633	–8%	–19%	3%
	90%	3,543	0.659	0.613	0.673	0.634	–8%	–18%	4%

^a^ The size of the resampled historical cohorts is given as a percentage of the full historical cohort sample size (N = 39,209 warfarin initiators). ^b^ Number of events refers to the number of events corresponding to the follow-up strategy used for disease risk score development (column 1). This has been averaged across the 1,000 resampled cohorts of each size. ^c^ C-index among the AT experience of warfarin initiators in the resampled historical cohorts. This has been averaged across the 1,000 resampled cohorts of each size. ^d^ C-index among the AT experience of warfarin initiators in the concurrent cohort. This has been averaged across the 1,000 DRS models fit in the resampled cohorts of each size. ^e^ C-index among the AT experience of dabigatran initiators in the concurrent cohort. This has been averaged across the 1,000 DRS models fit in the resampled cohorts of each size. ^f^ C-index among the AT experience of all initiators in the concurrent cohort. This has been averaged across the 1,000 DRS models fit in the resampled cohorts of each size. ^g^ Pseudo-bias estimated with the dry-run analysis is a measure of prognostic balance. Here it is presented in terms of the percent bias in the hazard ratio. This has been averaged across the 1,000 DRS models fit in the resampled historical cohorts of each size. Confidence intervals are 95% empirical intervals based on the 2.5^th^ and 97.5^th^ percentile.

## Discussion

As-treated follow-up, which restricts observation to person-time accrued by subjects while continuously receiving their initial treatment, is a common strategy in comparative effectiveness and safety research of medical products. However, such limited follow-up may be problematic for the development of robust DRS models, which rely on large numbers of outcome events to accommodate many covariates. Developing DRS models under an ITT approach allows investigators to take advantage of more study outcomes in the historical cohort, potentially permitting more confounders to be adjusted for and reducing overfitting. The comparison of DRS models fit using a variety of follow-up strategies based on the dry-run analysis may be a viable approach to identify the model likely to control the most confounding, though this requires the estimation of a valid PS.

In an example comparative safety study of two oral anticoagulants, we found that issues of DRS model overfitting were more acute when the DRS was developed under AT than when it was developed under ITT, but that these differences did not necessarily lead to appreciably worse confounding control in the analysis using the full historical cohort. However, results from our resampling study suggest that substituting DRSs developed under various ITT strategies for those developed under AT may be advantageous when the number of outcomes in the development cohort is small, since ITT DRSs generally had less “pseudo-bias” than AT DRSs in dry run analyses, and since ITT DRS-adjusted estimates were consistently closer to the reference estimate than AT-adjusted estimates. However, these differences were small, and additional investigation should consider whether these results hold in alternate contexts.

Interestingly, we found that DRS models fit among historical warfarin initiators showed better discrimination and calibration among concurrent dabigatran initiators than among concurrent warfarin initiators. This may indicate channeling of sicker patients to warfarin once dabigatran became available, leading to a higher prevalence of unmeasured risk factors or contraindications, such as frailty, among concurrent warfarin initiators and thus better fit among dabigatran initiators. The consequences of such channeling on historical development of DRSs warrants further attention.

Throughout our empirical assessment, all methods of DRS adjustment were biased toward the crude by between 6 percent and 9 percent compared to the reference PS-stratified estimate. While there are several reasons PS- and DRS-adjusted treatment effect estimates might differ, the consistent direction of bias observed here suggests that DRS methods were subject to more residual confounding than PS methods. This is consistent with previous studies of DRS development in the anticoagulant setting [23]. More empirical research is needed to determine the comparability of PS and DRS-based confounding control in settings where both are possible.

This study may be limited by reliance on results from a single empirical assessment. It is possible that the use of ITT DRSs to adjust AT effect estimates may lead to substantially less confounding control than was observed here in settings with different mechanisms of treatment discontinuation. Likewise, it should be noted that the reference PS-stratified treatment effect is not known to be true, and thus may not provide the ideal benchmark against which to compare DRS-adjusted estimates. However, previous clinical trials [24] and observational studies [25,26,27,28] indicate similar effects of dabigatran vs. warfarin on the risk of major bleeding events.

We advise serious consideration of the assumptions inherent in DRS adjustment before choosing the method of DRS development. In choosing to control confounding of an AT contrast with a DRS developed under ITT, we must assume that the ITT DRS ranks patients in roughly the same order as a correctly specified, robust AT DRS would. An example of when this assumption may not be tenable is if some covariates exert broadly differential effects when adherent or non-adherent to the comparator treatment. For example, if diabetes is not itself a risk factor for bleeding, but diabetics may be at a higher risk of warfarin-related bleeding, then the DRS model coefficient for diabetes may be null under ITT but positive under AT. This may adversely affect confounding control with the ITT DRS if many patients have diabetes, diabetes dramatically increases the risk of warfarin-related bleeding, and many patients discontinue warfarin soon after initiation, since the comparative ranking of patients with respect to bleeding risk may be completely altered.

In any analysis that censors patients based on treatment discontinuation, consideration of informative censoring is important. If treatment discontinuation, and thus censoring, is prognostic for the study outcome, a Cox proportional hazards model will produce biased estimates of the survival function [29]. The direction of this bias will correspond to the direction of the correlation between censoring and failure, and the degree of bias will correspond to the prevalence of censoring. This in itself will not adversely affect DRS-based confounding control since subjects will still be ranked appropriately, but if censoring is differential with respect to the confounders, the model coefficients themselves may be biased in ways that change the ranks [30]. Therefore, we might assume that development of a DRS model under AT follow-up will yield the worst confounding control when censoring is informative, common, and differential with respect to important confounders. In practice, censoring patients upon discontinuation of treatment may also induce selection bias in the treatment effect estimate. While methods to address selection bias exist [20,21], we did not make use of them in this example study. This should not be problematic, as all hazard ratios were estimated in the same AT population and therefore are subject to the same degree of selection bias, with differing amounts of residual confounding attributable to method of DRS adjustment.

Additional research is needed to explore alternate methods of DRS development and use for the purpose of confounding control. Potential alternate strategies for increasing DRS power might include obtaining additional historical data from increasingly early time periods, broadening cohort eligibility criteria to include similar patients who did not initiate the comparator treatment, or reducing the dimensionality of the DRS model in a principled manner. As with altering the follow-up approach for DRS development, each of these strategies also carries disadvantages. For example, developing DRSs in a historical cohort followed from long before introduction of the treatment of interest increases the likelihood of secular changes in covariate measurement (e.g., due to coding practices). Broadening eligibility for the DRS development cohort may increase the quantity of available events, but may also allow the inclusion of patients who failed to initiate the comparator treatment due to unmeasured contraindications. Likewise, reducing a DRS model’s dimensionality may require the omission of important confounders. Likewise, methods of DRS adjustment other than stratification, including regression adjustment with smooth functional forms and matching, as well as use of the DRS as a probability as opposed to a linear predictor, warrant further attention.

In conclusion, development of the DRS without censoring patients based on discontinuation may be a reasonable approach to reduce overfitting and enhance confounding control when DRS adjustment of AT effects is desired. This example may motivate further study in alternate contexts, which can help establish general recommendations for DRS development.

## Additional Files

The additional files for this article can be found as follows:

10.5334/egems.254.s1 Appendix 1. Details on the dry run analysis.

## References

[1] Miettinen, OS. Stratification by a multivariate confounder score. Am J Epidemiol. 1976; 104: 609–620. DOI: 10.1093/oxfordjournals.aje.a112339 998608

[2] Hansen, BB. The prognostic analogue of the propensity score. Biometrika. 2008; 95: 481–488. DOI: 10.1093/biomet/asn004

[3] Arbogast, PG and Ray, WA. Use of disease risk scores in pharmacoepidemiologic studies. Stat Methods Med Res. 2009; 18: 67–80. DOI: 10.1177/0962280208092347 18562398

[4] Arbogast, PG and Ray, WA. Performance of Disease Risk Scores, Propensity Scores, and Traditional Multivariable Outcome Regression in the Presence of Multiple Confounders. Am J Epidemiol. 2011; 174: 613–620. DOI: 10.1093/aje/kwr143 21749976

[5] Arbogast, PG, Kaltenbach, L, Ding, H and Ray, WA. Adjustment for Multiple Cardiovascular Risk Factors Using a Summary Risk Score. Epidemiology. 2008; 19: 30–37. DOI: 10.1097/EDE.0b013e31815be000 18091000

[6] Cadarette, SM, Gagne, JJ, Solomon, DH, Katz, JN and Sturmer, T. Confounder summary scores when comparing the effects of multiple drug exposures. Pharmacoepidemiol Drug Saf. 2010; 19: 2–9. DOI: 10.1002/pds.1845 19757416 PMC2800174

[7] Glynn, RJ, Gagne, JJ and Schneeweiss, S. Role of disease risk scores in comparative effectiveness research with emerging therapies: Disease Risk Scores for Emerging Therapies. Pharmacoepidemiol Drug Saf. 2012; 21: 138–147. DOI: 10.1002/pds.3231 22552989 PMC3454457

[8] Tadrous, M, Gagne, JJ, Stürmer, T and Cadarette, SM. Disease risk score as a confounder summary method: systematic review and recommendations: DRS as a Confounder Summary Method. Pharmacoepidemiol Drug Saf. 2013; 22: 122–129. DOI: 10.1002/pds.3377 23172692 PMC3691557

[9] Leacy, FP and Stuart, EA. On the joint use of propensity and prognostic scores in estimation of the average treatment effect on the treated: a simulation study. Stat Med. 2014; 33: 3488–3508. DOI: 10.1002/sim.6030 24151187 PMC3995901

[10] Stuart, EA, Lee, BK and Leacy, FP. Prognostic score–based balance measures can be a useful diagnostic for propensity score methods in comparative effectiveness research. J Clin Epidemiol. 2013; 66: S84–S90.e1 DOI: 10.1016/j.jclinepi.2013.01.013 23849158 PMC3713509

[11] Hansen, BB. Bias reduction in observational studies via prognosis scores. Technical Report 441, University of Michigan, Statistics Department, 2006 Available at: http://dept.stat.lsa.umich.edu/~bbh/rspaper2006-06.pdf. Accessed March 24, 2016.

[12] Harrell, FE, Lee, KL, Califf, RM, Pryor, DB and Rosati, RA. Regression modelling strategies for improved prognostic prediction. Stat Med. 1984; 3: 143–152. DOI: 10.1002/sim.4780030207 6463451

[13] Hernan, MA and Hernandez-Diaz, S. Beyond the intention-to-treat in comparative effectiveness research. Clin Trials. 2012; 9: 48–55. DOI: 10.1177/1740774511420743 21948059 PMC3731071

[14] Harrell, FE, Lee, KL and Mark, DB. Tutorial in biostatistics multivariable prognostic models: issues in developing models, evaluating assumptions and adequacy, and measuring and reducing errors. Stat Med. 1996; 15: 361–387. DOI: 10.1002/(SICI)1097-0258(19960229)15:4<361::AID-SIM168>3.0.CO;2-4 8668867

[15] Schneeweiss, S, Rassen, JA, Glynn, RJ, Avorn, J, Mogun, H and Brookhart, MA. High-dimensional Propensity Score Adjustment in Studies of Treatment Effects Using Health Care Claims Data. Epidemiology. 2009; 20: 512–522. DOI: 10.1097/EDE.0b013e3181a663cc 19487948 PMC3077219

[16] Pisters, R, Lane, DA, Nieuwlaat, R, de Vos, CB, Crijns, HJGM and Lip, GYH. A novel user-friendly score (HAS-BLED) to assess 1–year risk of major bleeding in patients with atrial fibrillation: the Euro Heart Survey. Chest. 2010; 138: 1093–1100. DOI: 10.1378/chest.10-0134 20299623

[17] Lip, GYH, Nieuwlaat, R, Pisters, R, Lane, DA and Crijns, HJGM. Refining clinical risk stratification for predicting stroke and thromboembolism in atrial fibrillation using a novel risk factor-based approach: the euro heart survey on atrial fibrillation. Chest. 2010; 137: 263–272. DOI: 10.1378/chest.09-1584 19762550

[18] Gage, BF, van Walraven, C, Pearce, L, et al. Selecting patients with atrial fibrillation for anticoagulation: stroke risk stratification in patients taking aspirin. Circulation. 2004; 110: 2287–2292. DOI: 10.1161/01.CIR.0000145172.55640.93 15477396

[19] Wyss, R, Lunt, M, Brookhart, MA, Glynn, RJ and Stürmer, T. Reducing Bias Amplification in the Presence of Unmeasured Confounding through Out-of-Sample Estimation Strategies for the Disease Risk Score. J Causal Inference. 2014; 2 DOI: 10.1515/jci-2014-0009 PMC4193945 25313347

[20] Harrell, FE, Califf, RM, Pryor, DB, Lee, KL and Rosati, RA. Evaluating the yield of medical tests. Jama. 1982; 247: 2543–2546. DOI: 10.1001/jama.1982.03320430047030 7069920

[21] Demler, OV, Paynter, NP and Cook, NR. Tests of calibration and goodness-of-fit in the survival setting. Stat Med. 2015; 34: 1659–1680. DOI: 10.1002/sim.6428 25684707 PMC4555993

[22] Wyss, R, Hansen, BB, Ellis, AR, et al. The “Dry-Run” Analysis: A Diagnostic for Evaluating Risk Scores for Confounding Control. Am J Epidemiol. (In press). 10.1093/aje/kwx032 PMC5411679 28338910

[23] Kumamaru, H, Gagne, JJ, Glynn, RJ, Setoguchi, S and Schneeweiss, S. Comparison of high-dimensional confounder summary scores in comparative studies of newly marketed medications. J Clin Epidemiol. 2016; 76: 200–208. DOI: 10.1016/j.jclinepi.2016.02.011 26931292

[24] Connolly, SJ, Ezekowitz, MD, Yusuf, S, et al. Dabigatran versus warfarin in patients with atrial fibrillation. N Engl J Med. 2009; 361: 1139–1151. DOI: 10.1056/NEJMoa0905561 19717844

[25] Yao, X, Abraham, NS, Sangaralingham, LR, et al. Effectiveness and Safety of Dabigatran, Rivaroxaban, and Apixaban Versus Warfarin in Nonvalvular Atrial Fibrillation. J Am Heart Assoc. 2016; 5 DOI: 10.1161/JAHA.116.003725 PMC4937291 27412905

[26] Abraham, NS, Singh, S, Alexander, GC, et al. Comparative risk of gastrointestinal bleeding with dabigatran, rivaroxaban, and warfarin: population based cohort study. BMJ. 2015; 350: h1857 DOI: 10.1136/bmj.h1857 25910928 PMC4413863

[27] Larsen, TB, Rasmussen, LH, Skjøth, F, et al. Efficacy and Safety of Dabigatran Etexilate and Warfarin in “Real-World” Patients With Atrial Fibrillation. J Am Coll Cardiol. 2013; 61: 2264–2273. DOI: 10.1016/j.jacc.2013.03.020 23562920

[28] Lauffenburger, JC, Farley, JF, Gehi, AK, Rhoney, DH, Brookhart, MA and Fang, G. Effectiveness and Safety of Dabigatran and Warfarin in Real-World US Patients With Non-Valvular Atrial Fibrillation: A Retrospective Cohort Study. J Am Heart Assoc. 2015; 4: e001798 DOI: 10.1161/JAHA.115.001798 25862791 PMC4579955

[29] Tsiatis, A. A nonidentifiability aspect of the problem of competing risks. Proc Natl Acad Sci U S A. 1975; 72: 20–22. DOI: 10.1073/pnas.72.1.20 1054494 PMC432231

[30] Huang, X and Zhang, N. Regression Survival Analysis with an Assumed Copula for Dependent Censoring: A Sensitivity Analysis Approach. Biometrics. 2008; 64: 1090–1099. DOI: 10.1111/j.1541-0420.2008.00986.x 18266895 PMC4037927

