# Supplementary material for: Controlling Confounding in a Study of Oral Anticoagulants: Comparing Disease Risk Scores Developed Using Different Follow-Up Approaches
Source: EGEMS (Wash DC). 2019 Jul 15;7(1):27. doi: 10.5334/egems.254 (PMC6640656; doi:10.5334/egems.254)
Supplement: Appendix 1. — Details on the dry run analysis. [file egems-7-1-254-s1.pdf]

Appendix 1: details on the dry run analysis

The "dry run analysis" is a procedure described by Hansen (2006) to check that a disease risk score (DRS) induces prognostic balance. The procedure is performed as follows. For the purposes of description, consider A to be an indicator for treatment status, where $A = 1$ indicates active treatment and $A = 0$ indicates the comparator treatment. Also, let L denote the vector of confounders of the relationship between A and the study outcome.

The basic idea is to use a propensity score to randomly assign a "pseudo-treatment" value, say A^*, for all subjects with $A = 0$, then estimate a treatment effect comparing $A^* = 1$ vs. $A^* = 0$, conditional on the DRS. If the DRS induces prognostic balance and the propensity score is correctly specified, then the treatment effect comparing $A^* = 1$ and $A^* = 0$ must be null since both groups are *actually* untreated. The divergence of this treatment effect from its null value is called the "pseudo-bias" and may be thought of as a measure of the degree to which the DRS induces prognostic balance. In practice, the process of randomly assigning values for A^* and estimating the pseudo-bias must be repeated a large number of times, so that an average "pseudo-bias" (and its corresponding confidence interval) can be obtained. Below is the algorithm we used to obtain estimates of the pseudo-bias and its CI.

1. Begin with the study population in which treatment effects will be estimated
    a. Estimate a valid PS model and obtain PS values for all subjects in the population, call the PS value for subject i PS_i

   b. Obtain the prevalence of treatment $p_A = pr(A = 1)$ in the study population. This will be used to adjust the PS so that the prevalence of pseudo-treatment is approximately equal to the prevalence of actual treatment.

2. Restrict the study population to those who initiated the comparator treatment ($A = 0$, in this case these are the warfarin initiators)
3. Generate the probability to be used for randomly drawing pseudo-treatment values
   a. To obtain the same treatment prevalence in the pseudo-population as in the study population, the PS must be adjusted by a constant c, which can be found by solving the equation

$$p_A = \frac{1}{N}\sum_{i=1}^{N}\frac{1}{1 + e^{-(c+logit(PS_i))}}$$

   b. Use c to obtain the probability of pseudo-treatment PS_i^*

$$PS_i^* = \frac{1}{1 + e^{-(logit(PS_i)+c)}}$$

4. Randomly assign the pseudo-treatment status A^* with probability PS_i^*

$$A^* \sim Bernoulli(PS_i^*)$$

5. Model the DRS
   a. If the DRS is to be estimated among those in the study population with $A = 0$, model the DRS among those with $A^* = 0$ in the pseudo-population and assign DRS values to those with $A^* = 1$ and $A^* = 0$.

b. If the DRS is to be estimated in an external population (e.g., a historical population of comparator drug initiators), model the DRS in that population and assign DRS values to those with $A^* = 1$ and$A^* = 0$.

6. Estimate a treatment effect comparing $A^* = 1$ and $A^* = 0$, conditional on the DRS. In our example, we used cox proportional hazards models with stratification on deciles of the DRS (indexed by s) to estimate the effect of pseudo-treatment on the log hazard ratio scale as $\hat{\beta}_1$ in the model

$$h(t|A^*, A = 0) = h_{0,s}(t)e^{\hat{\beta}_1 A^*}$$

Note: Other choices of effect estimation method and DRS adjustment method are of course possible. Such methods should be chosen to match those used in the main study. For example, if the DRS in question will be used for matching, the dry run procedure should use matching as well. Likewise, if the effect measure of interest is the odds ratio estimated via logistic regression, the dry run procedure should use logistic regression as well. In our case, we used Cox proportional hazards models to estimate hazard ratios, with stratification on deciles of the DRS.

7. Repeat steps 3 through 5 a large number (e.g., 1000) times to obtain a distribution for the pseudo-bias$\hat{\beta}_1$. The average pseudo-bias (and its empirical 2.5^{th} and 97.5^{th} percentiles) can then be reported as a measure of prognostic balance, with precise estimates of null values indicating good performance. In our case, reporting was

done on the percent bias in hazard ratio scale, so that the pseudo bias PB was averaged over the $j = \{1,2,\ldots,1{,}000\}$ resamplings as:

$$PB = \left(\exp\left\{\frac{1}{1{,}000}\sum_{j=1}^{1{,}000}\hat{\beta}_{1,j}\right\} - 1\right) * 100\%$$
